# The water-land-food nexus of first-generation biofuels

**DOI:** 10.1038/srep22521

**Published:** 2016-03-03

**Authors:** Maria Cristina Rulli, Davide Bellomi, Andrea Cazzoli, Giulia De Carolis, Paolo D’Odorico

**Affiliations:** 1 Departiment of Civil and Environmental Engineering, Politecnico di Milano, Piazza Leonardo da Vinci 32, I-20133 Milan, Italy; 2 Department of Environmental Sciences, University of Virginia, 291 McCormick Rd., Charlottesville, VA 22903, USA; 3 National Socio-Environmental Synthesis Center, University of Maryland, Annapolis, MD 21401, USA.

## Abstract

Recent energy security strategies, investment opportunities and energy policies have led to an escalation in biofuel consumption at the expenses of food crops and pastureland. To evaluate the important impacts of biofuels on food security, the food-energy nexus needs to be investigated in the context of its linkages with the overall human appropriation of land and water resources. Here we provide a global assessment of biofuel crop production, reconstruct global patterns of biofuel crop/oil trade and determine the associated displacement of water and land use. We find that bioethanol is mostly produced with domestic crops while 36% of biodiesel consumption relies on international trade, mainly from Southeast Asia. Altogether, biofuels rely on about 2-3% of the global water and land used for agriculture, which could feed about 30% of the malnourished population. We evaluate the food-energy tradeoff and the impact an increased reliance on biofuel would have on the number of people the planet can feed.

## Introduction

**Figure 1:**
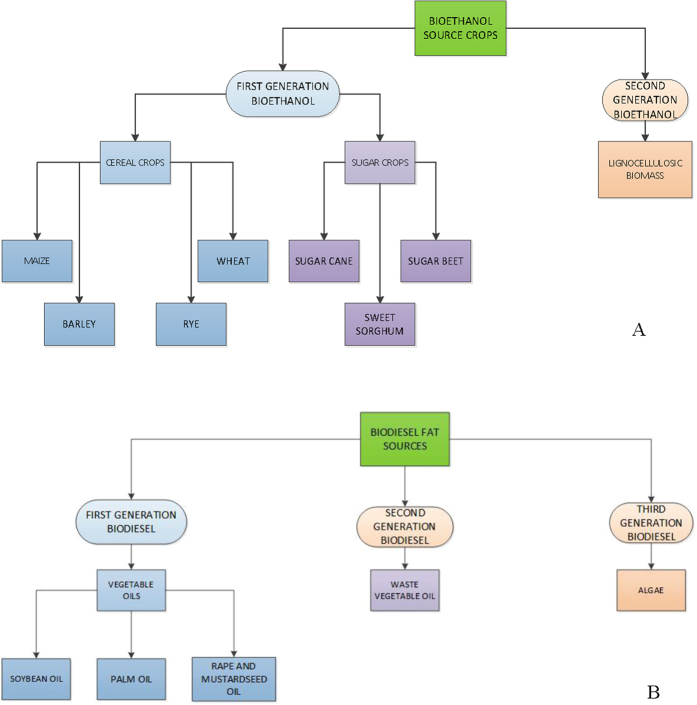
(**A**) Bioethanol is obtained from carbohydrates of sugar or starchy crops via alcoholic fermentation, a biological process in which bacteria convert sugars such as glucose, fructose and sucrose into ethanol. (**B**) Biodiesel is a vegetable oil or animal fat based fuel; it consists of long-chain alkyl (methyl, ethyl, or propyl) esters. It is typically made by chemically reacting lipids with an alcohol, which leads to the production of fatty acid esters. This chemical reaction is known as trans-esterification.

The synthesis of biofuels from plant biomass (mostly crops) offers the opportunity to rely on energy from geologically recent carbon as an alternative to fossil fuel^1^. The two main types of biofuels used for transportation are bioethanol and biodiesel. The former is made from sugar and starchy crops (Fig. 1A) and can be blended with gasoline, while the latter is produced using organic fats and vegetable oils (Fig. 1B) and can be blended with petrol diesel^1^.

In recent years, rising interest in biofuel production has resulted both from the increase in oil prices and new U.S. and E.U. energy policies mandating a certain degree of reliance on renewable energy as a strategy to curb greenhouse gas (GHG) emissions from the transport sector^2^,^3^,^4^. Biofuels may contribute to the enhancement of energy security in countries lacking direct access to fossil fuel deposits, the reduction of greenhouse gas (GHG) emissions, and a more profitable use of crops than in the food market where the same agricultural products would often be less valued.

The production of biofuel crops, however, can also have negative impacts on the environment, particularly through land use change and deforestation^5^,^6^,^7^,^8^,^9^,^10^. Moreover, biofuels require water and land resources^11^,^12^ that could otherwise be used for the production of food^13^,^14^ and ecosystem goods and services. Therefore, the competing needs for land and water resources by food and biofuel production are at the forefront of the energy-food debate^15^,^16^, which is fueled by recent food crises and associated spikes in food prices^17^,^13^,^4^. As a result, a number of outstanding questions on the energy-food nexus have arisen, including the number of people who could be fed by the crops used for biofuels; the extent to which these crops, if used for food consumption in the producing countries, could alleviate malnutrition; and whether bioenergy production entails an important displacement of land use^18^ through its reliance on the trade of feedstock or vegetable oil.

Between 2000 and 2008 the consumption of alcohol for non-food uses (“other uses”, the FAO data sets)^19^ (i.e., bioethanol) more than doubled in the USA and underwent a five-fold increase in Brazil, concurrently with the global increase in bioethanol consumption reported by OECD/FAO^20^. Overall, in 2013 about 86 million tons of biofuels were consumed globally, including 65 million tons of bioethanol and 21 of biodiesel. We estimate that in 2013 1.91 × 10^6^ TJ/y of bioethanol and 0.82 × 10^6^ TJ/y of biodiesel energy were produced worldwide (Table 1), claiming an area of about 41.3 million ha, which accounts for about 4% of the global arable area, consistent with findings by^14^. Biofuel production consumed 216 billion m^3^ of water, which corresponds to about 3% of the global water consumption for food production^21^. Our results also show that, while the water footprint of biodiesel and bioethanol energy are overall comparable, the land footprint of biodiesel is on average more than 100% greater than that of bioethanol (Table 2). These values, however, vary substantially, depending on the crop and geographic location.[Table t3]

### Bioethanol

**Figure 2:**
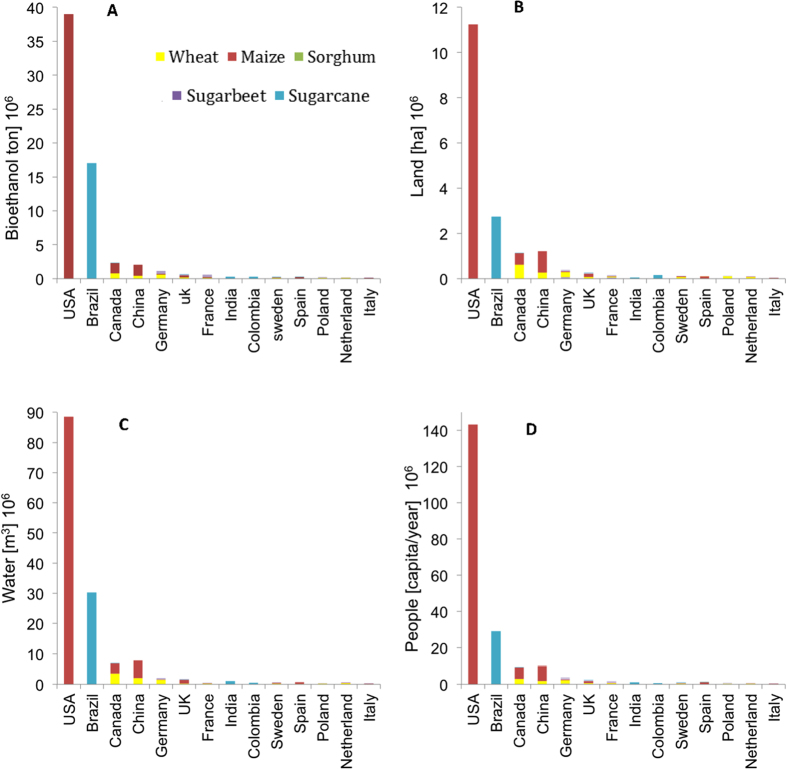
For the top 14 bioethanol consumers we show the resources used for bioethanol production (**A**), including both domestic production for in country use and imports) in terms of: (**B**) Land; (**C**) Water; (**D**) Food equivalent, i.e., people who could be fed with crops used for bioethanol (based on country-specific rates of calorie consumption (Table S2)). Most of the global water consumption for bioethanol production (>50%) is contributed by maize in the USA and sugar cane in Brazil (**C**). Because of their reliance on these two different feedstocks, the water and land used in Brazil are substantially lower than in the USA (Table 2). The water consumed globally for bioethanol is primarily from rainwater (or “green”) (76%), though considerable amounts of (“grey”) water for pollutant dilution (14%) and irrigation (“blue”) water (10%) are also used (Table 3).

Our findings show that bioethanol is produced mostly with sugarcane and maize followed by wheat, sugarbeet and sorghum (Fig. S1A). Because of its higher ethanol yield, maize accounts for 67% of the global bioethanol supply (Fig. S1B). However, while sugarcane is by far the highest contributor to bioethanol production (in terms of crop biomass), it is not the greatest water consumer because bioethanol produced from maize and wheat has a greater water footprint^11^,^12^ (Fig. S1C). Thus the different bioethanol crops used by producing countries explain their different use of resources (water, land, and food equivalent) (Fig. 2). The impact of bioethanol production is also evaluated in terms of the number of people who could be fed by bioethanol crops (Fig. 2D). We find that about 200 million people could be fed by the agricultural resources used to meet the bioethanol demand in the countries listed in Table 1.

### Biodiesel

**Figure 3:**
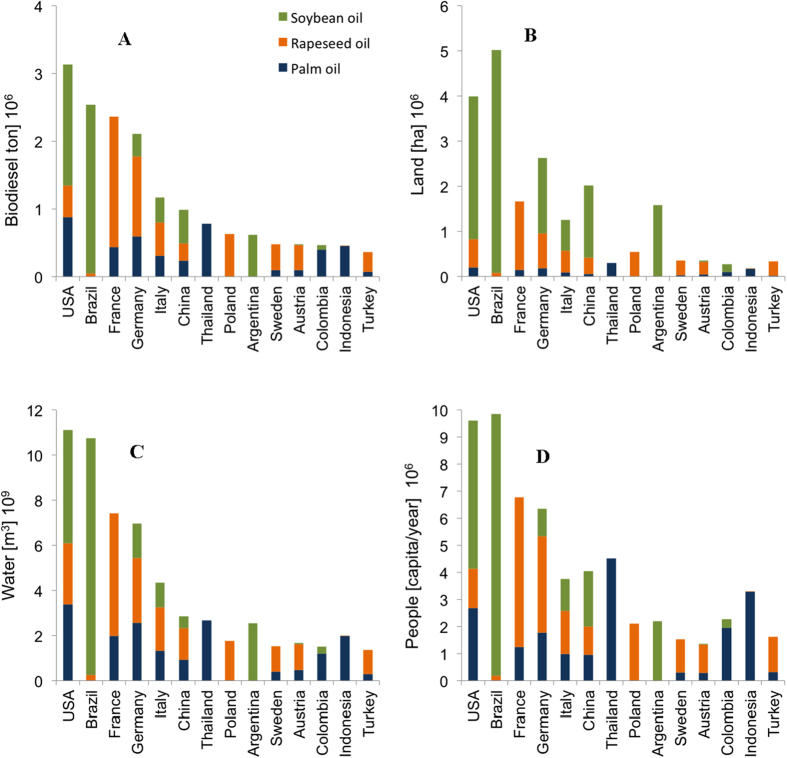
Biodiesel consumption (**A**). Land (**B**) and Water (**C**) used for biodiesel production, and (**D**) Number of People who could be fed with the crops used for biodiesel production in the top 14 biodiesel consumers in the world (based on country-specific rates of calorie consumption (Table S2)).

Biodiesel is produced in equal proportions with rapeseed, soybean and palm oil (Fig. S2A). These oils have comparable biodiesel yields, but different extraction rate (i.e., crop oil yield) resulting in the consumption of a double amount of soybean compared to rapeseed (Fig. S2B). The proportion of water consumed by each of these vegetable oils, however, is not the same: biodiesel produced with palm oil is the most water demanding^11^,^12^ (Fig. 3). Most of the global consumption of biodiesel takes place in OECD + EU27 countries (listed in the caption of Table S1).

The greatest biodiesel consumers are USA and Brazil, followed by France, Germany, and Italy (Fig. 3A). These countries (USA, France, Germany and Italy) rely mostly on rape-mustard seed and soybean oil (and, in smaller amounts, palm oil), as do most of the other OECD + EU27 countries. Different oil consumption patterns are found in Brazil, which strongly relies on soybean oil. Countries that rely more on soybean seed oil use (either domestically or internationally) more land per unit energy consumed (Table 2; Fig. 3B). Because oil palm is a very high-yield crop, soybean oil and rape-mustard seed oil consumption are the main contributors to the land footprint of biodiesel energy (Table 2; Fig. 3B). An analysis based on per capita calorie requirements and the caloric content of biodiesel crops shows that about 70 million people could be fed by the food calories of the vegetable oils used for biodiesel production in the top 29 consumer countries that account for 97% of the global biodiesel consumption (Table 1; Fig. 3D).

The amounts of feedstock determined with the approach used in this study are in agreement with those reported by other sources. For example ANP^22^ reports the use of 2,041 × 10^3^ m^3^ of soybean oil for biodiesel consumption in Brazil, while the data sources and methods used in this paper lead to an estimate of 2,480 × 10^3^ m^3^. In the case of Europe, USDA-GAIN^23^ reports for rape-seed oil a consumption of 5,770 × 10^3^ m^3^, which favorably compares with our estimate of 6,097 × 10^3^ m^3^, while for soybean oil USDA-GAIN reports 850 × 10^3^ m^3^, in overall agreement with our estimate 1060 × 10^3^ m^3^. Likewise, USDA-GAIN reports a combined estimate for palm oil and used cooking oil of 2,920 × 10^3^ m^3^, while our analyses show a value of 3,425 × 10^3^ m^3^. There is an overall agreement among these sources within a 10–20% tolerance.

On average, biodiesel requires about the same amount of water resources but more land than bioethanol (Table 2). The direct impact of biodiesel on food security is similar to that of bioethanol, if evaluated in terms of number of people who could be fed per unit of biofuel energy (Table 2). However, because the global production of biodiesel is overall smaller than that of bioethanol, the impact of bioethanol on the number of people who could be fed is greater (Figs 2D and 3D).

### Dependence on international trade

**Figure 4:**
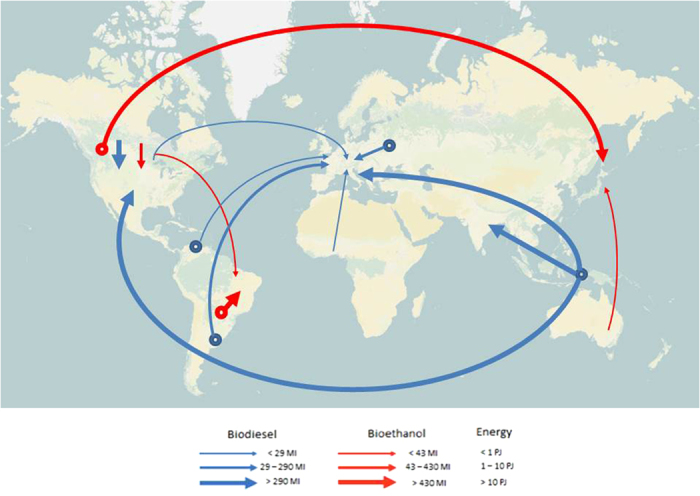
World map of energy flows related to bioethanol and biodiesel trade. The round symbol refers to multiple countries in the area (1PJ = 10^15^J; 1 Ml = 10^6^ litres). [Figure generated with ®Microsoft PowerPoint. The base map is available from OpenStreetMap (http://www.openstreetmap.org/copyright) and is licensed under the Attribution-Share-Alike 2.0 license. The license terms can be found on the following link: http://creativecommons.org/licenses/by-sa/2.0/].

The natural resources used for biofuel production are partly available domestically in the country where the biofuel is consumed and partly (virtually) imported from other countries that produce and export feedstock for bioenergy. Globally, 97% of the water footprint and 96% of the land footprint of bioethanol production are internal. For the external portion of these footprints the associated global patterns of trade are dominated by Japan’s imports from the U.S.A. and Australia and trade partnerships internal to South America (Fig. 4).

In the case of biodiesel we were able to trace imports only for the aggregate of OECD/EU27 countries rather than for each country individually (see Supplementary Information). For this group of countries, 59% of the water footprint and 80% of the land footprint of biodiesel were internal. Thus, while most of land used to produce bioethanol is internal to the countries where it is consumed, in the case of biodiesel there is a stronger reliance on trade. However, even though bioethanol imports are still just a fraction of the global production, the energy flows associated with biodiesel trade are only about five times those for bioethanol because of the overall greater worldwide consumption of bioethanol (Fig. 4). The major energy flows related to biodiesel are from Malaysia, Indonesia and Papua New Guinea because of palm oil trade. There are also other important flows from South America (soybean and rape-mustard seed oil) and Canada, (rape-mustard seed oil) (Fig. 4). The European Union is the biggest importer (Fig. 4). Most of the virtual water trade (75%) associated with the biodiesel market is contributed by palm oil, while the virtual water trade of mustard and rapeseed oil tends to occur within the OECD + EU27 country group and cannot be resolved by our analysis (see Supplementary Materials).

The environmental impacts of European palm oil imports from Malaysia and Indonesia (Fig. 4 and S3) have been highlighted by a number of recent studies. Such impacts include high deforestation rates and large carbon emissions in Malaysia and Indonesia due to oil palm plantations^8^,^5^ as well as losses of habitat and threats to biodiversity^24^. In response, the European Union has taken some action to limit these unwanted effects on the environment^25^. For instance, biofuels produced from feedstocks grown on land with “high biodiversity value” (e.g., primary forests, peatlands, wetlands, certain woodlands and grassland) are not accepted under E.U. renewable energy mandates. The direct and indirect effects of biofuel production on these ecosystems, however, remain difficult to verify^26^.

### The Water-Food-Energy Nexus

First generation bioethanol (i.e., produced from food crops) is still the major contributor to the global biofuel supply. The production of second and third generation biofuels from cellulosic plant tissues or algae is overall negligible (but is expected to be substantial in the coming 10–20 years^27^,^28^), despite their lower water and land footprints, and their lack of competition with food production.

In addition to the environmental impacts, biofuel production has important societal implications that can be better understood by examining the energy-food-water nexus of biofuels. Crops used to produce 1 TJ of biofuel would be sufficient to feed 110 and 90 people in the case of bioethanol and biodiesel, respectively. Interestingly, bioethanol production uses as feedstock major staple crops (e.g., maize and wheat) that could be directly used as food. In the case of biodiesel the competition with food is partly mitigated by the growing reliance of the biofuel industry on recycled cooking oil (up to 88% in the case of the U.K.^29^). At the global scale, we find that about 280 million people (i.e., more than one fourth of the malnourished population in the world^30^) could be fed with the crop calories used for biofuels in 2013. We stress, however, that this is not the number of people that would likely see an improvement in their access to food, should biofuel use be reduced to zero. Clearly, there are important economic and policy drivers underlying the current trends in biofuel consumption that are not accounted for in our 1:1 replacement of biofuel with food crops. Regardless, these numbers highlight the important contrast between biofuel production (which provides only 4 percent of energy needed by the transport sector and 0.2% of the global energy use in all sectors^31^), and food security (which could be strongly enhanced by biofuel crops). This fact calls for revisions to current climate change mitigation policies based on biofuels, as more recently recommended by the E.U.^25^. On April 2015 the European Parliament approved a reform of the Renewable Energy Directive (RED), which includes a 7 percent cap on food crop based biofuels for the transport sector.

The water-food-energy nexus of biofuel consumption can be further analyzed by evaluating the tradeoff between the maximum number of people the planet could feed, and a partial conversion of the societal metabolism from fossil fuel reliance to renewable energy^32^,^33^,^34^. With the industrial revolution, human societies switched from a metabolism based only on solar energy (i.e., photosynthesis) to an increasing reliance on fossil fuels (i.e., solar energy from a geological past)^33^. Thanks to this reliance on fossil resources, humans have been able to increase the agricultural production and greatly enhance their access to energy and food^35^. Biofuels offer a mechanism through which society could reduce its reliance on fossil fuels. Our study as well as recent analyses of global food security^14^,^36^, however, have shown that the global agricultural land could not be sufficient to meet the current human demand for food and energy. How many people can be supported by the food and bioenergy the planet can produce?Assuming a 10% reliance on biofuels (b = 0.10) (E.U., 2009) and using the bioenergy footprint values determined by this study, we find (see Methods) that the area A can meet the food and energy requirements of 6.7 billion people with the current average global food and energy demand. However, patterns of economic development show shifts toward higher energy consumption rates and more calorie demanding diets (e.g., more meat) as societies become more affluent^37^. To evaluate the impact of these increasing trends in food and energy demand, we recalculate the population size that could be sustained (100% food and 10% transport energy) by the same agricultural area, A, using average consumption rates characteristic of the E.U. (see Methods); in these conditions the population size would be P = 4.8 billion people, which would decrease to P = 4.4 billion people with b = 0.20 and P = 2.5 billion people with 100% reliance on biofuels for transport energy (b = 1).

Despite their being based only on average yields and consumption rates, these calculations allow us to relate population size to its food and energy demand, and dependency on fossil fuels. These results highlight how the societal reliance on fossil fuels cannot be reverted by first generation bioethanol without undermining the food security of human societies. It should be stressed that the competition between food and biofuels is expected to become even more intense in the near future, with the world’s population predicted to reach 9 billion by 2050.

The potential development of second and third generation biofuels is an important step in the direction of mitigating the food-biofuel competition through new technologies relying on agricultural waste.

## Methods

We use biofuel consumption data, inferred from the FAOSTAT database^38^, to determine the amounts and types of crops used for bioethanol and biodiesel production in each country or country group, while the total values of bioethanol and biodiesel production and consumption are taken from other sources (Table S1). Because the FAOSTAT database does not provide estimates of error or uncertainty, the degree of uncertainty around the estimates presented in this paper remains unknown. Our study reconstructs patterns of biofuel consumption and trade using FAO data^38^ and other reports (i.e. Eurostat database^39^, US Energy International Administration^40^, USDA-Foreign agricultural service-Global Agricultural Information Network,^41^ Epure^42^, UK Department for Transport^29^, French Environment and Energy Management Agency (ADEME)^43^, Swedish Energy Agency^44^, Italian Ministry of Economic Development^45^, Agência Nacional do Petróleo, Gás Natural e Biocombustíveis – ANP^46^), without assuming a percentage of biofuel blending with diesel or gasoline. It accounts for the effect of trade on the water and land footprint of biofuels and determines the internal and external portion of these footprints. Finally, it evaluates the extent to which biofuels can be used to reduce our societal reliance on fossil fuels, while maintaining levels of food production that are sufficient to meet the needs of the global population. For more details, see the Supplementary Materials.

## Additional Information

**How to cite this article**: Rulli, M. C. *et al.* The water-land-food nexus of first-generation biofuels. *Sci. Rep.* **6**, 22521; doi: 10.1038/srep22521 (2016).

## Supplementary Material

Supplementary Information

## Figures and Tables

**Table 1:** A summary of the biofuel energy consumed in each country during the year 2013, the associated consumption of water and cultivated land area, and the number of people who could be fed by the food calories used for biofuel production considering the diets of the consumer (^1^) and producer (^2^) countries respectively.

		Biofuel energy consumed (10^3^ TJ/yr)	Water consumed for biofuel (10^6^ m^3^/yr)	Area cultivated for biofuel (10^3^ ha)	People^1^ 10^6^ (−)	People^2^ 10^6^ (−)
Bioethanol	USA	1162.4	88498.6	11245.4	143.3	147.9
	Brazil	506.7	30254.6	2752.0	29.1	28.6
	Canada	69.3	6853.5	1127.7	9.3	8.8
	China	62.0	7744.6	1212.1	10.0	8.8
	Germany	32.0	1960.8	331.5	3.4	3.7
	UK	19.2	1718.1	237.9	2.1	2.1
	France	16.6	694.4	122.6	1.6	1.9
	India	9.0	1097.4	60.7	1.0	1.0
	Colombia	8.6	505.5	160.5	0.6	0.4
	Sweden	7.6	598.6	106.4	0.8	0.8
	Spain	7.1	665.5	95.1	1.0	1.0
	Poland	6.0	387.5	99.3	0.7	0.7
	Netherlands	5.0	593.0	93.6	0.6	0.6
	Italy	3.1	229.2	39.6	0.4	0.4
	**Total**	**1914.7**	**141801.4**	**17684.4**	**203.9**	**206.7**
Biodiesel	USA	125.9	11105.5	3990.4	9.6	11.1
	Brazil	101.9	10741.1	5018.2	9.9	9.9
	France	94.9	7414.3	1664.1	6.8	7.5
	Germany	84.8	6956.0	2626.6	6.4	7.3
	Italy	46.9	4339.1	1253.9	3.8	4.2
	China	39.7	2848.4	2015.3	4.0	4.3
	Thailand	31.3	2679.2	297.6	4.5	4.5
	Spain	31.0	3446.3	432.4	2.8	4.1
	Poland	25.3	1754.3	544.2	2.1	2.1
	UK	25.1	2589.5	271.2	2.0	3.2
	Argentina	24.7	2542.9	1585.4	2.2	2.2
	Sweden	19.2	1517.4	353.7	1.5	1.7
	Austria	19.1	1665.4	355.5	1.4	1.5
	Colombia	18.7	1502.1	270.0	2.3	2.6
	Indonesia	18.2	1980.3	175.6	3.3	2.6
	Turkey	14.7	1362.9	331.4	1.6	1.7
	Belgium	12.5	1036.0	234.1	0.9	1.1
	Portugal	11.1	1158.9	529.7	0.8	0.9
	Netherlands	10.8	1024.6	174.7	0.8	1.3
	Canada	10.7	1116.3	318.1	0.9	1.0
	Peru	9.9	678.9	90.6	1.6	1.4
	Denmark	9.6	877.2	109.3	0.7	1.0
	Czech Rep.	9.2	921.0	202.2	0.8	0.8
	Finland	8.9	820.7	175.5	0.6	0.8
	Romania	6.2	850.1	234.8	0.5	0.6
	Greece	5.8	598.0	206.0	0.5	0.6
	Malaysia	4.1	396.8	38.1	0.5	0.6
	Slovakia	3.4	340.5	85.7	0.3	0.3
	India	1.9	198.3	40.0	0.4	0.3
	**Total**	**825.4**	**74462.2**	**23624.3**	**73.5**	**81.3**
	**Grand Total**	**2740.1**	**216263.5**	**41308.8**	**277.4**	**288.0**

We concentrate on the top 14 bioethanol consumers (≈85% of global consumption) and top 29 biodiesel consumers (≈81% of global consumption; about 98% of the global biodiesel consumption is consumed by 46 countries).

**Table 2:** Water and land needed to produce one TJ of energy used in the top consuming countries during the year 2013, and the number of people that could be fed by the associated bioethanol crops, based on the diets of the consumer (^1^) and producer (^2^) countries, respectively.

		10^3^ m^3^/TJ	ha/TJ	cap^1^/TJ	cap^2^/TJ
Bioethanol	USA	76	10	123	127
	Brazil	60	5	57	56
	Canada	99	16	134	126
	China	125	20	162	142
	Germany	61	10	105	116
	UK	89	12	112	112
	France	42	7	97	113
	India	122	7	112	107
	Colombia	59	19	72	49
	Sweden	79	14	110	110
	Spain	94	13	145	140
	Poland	64	16	116	116
	Netherlands	118	19	114	114
	Italy	73	13	126	130
	*Mean*	**82**	**13**	**113**	**111**
	*Weighted mean*	**74**	**9**	**106**	**108**
Biodiesel	USA	88	32	76	88
	Brazil	105	49	97	97
	France	78	18	71	79
	Germany	82	31	75	87
	Italy	88	25	76	90
	China	72	51	102	109
	Thailand	86	10	145	145
	Spain	111	14	90	134
	Poland	69	22	84	84
	UK	103	11	79	128
	Argentina	103	64	89	89
	Sweden	79	18	80	87
	Austria	87	19	71	79
	Colombia	80	14	122	141
	Indonesia	109	10	182	145
	Turkey	93	23	111	116
	Belgium	83	19	72	86
	Portugal	105	48	75	84
	Netherlands	95	16	78	117
	Canada	104	30	85	98
	Peru	68	9	161	145
	Denmark	91	11	69	103
	Czech Rep	100	22	87	87
	Finland	92	20	72	87
	Romania	138	38	82	92
	Greece	102	35	84	101
	Malaysia	98	9	120	145
	Slovakia	100	25	100	102
	India	104	21	189	150
	*Mean*	**91**	**24**	**95**	**106**
	*Weighted mean*	**90**	**29**	**89**	**99**

The weighted means is calculated with respect to the amounts of energy consumed by each country.

**Table 3 t3:** Green, Blue, Grey water footprint components of bioethanol and biodiesel energy in the major consuming countries.

		Green	Blue	Grey	Total
		(m^3^/GJ)	(m^3^/GJ)	(m^3^/GJ)	(m^3^/GJ)
Bioethanol	USA	52.3	6.3	17.6	76.1
	Brazil	53.2	2.2	4.4	59.7
	Canada	80.6	1.8	16.5	98.9
	China	81.6	14.4	28.9	124.8
	Germany	46.1	1.0	14.1	61.2
	UK	73.7	5.4	10.2	89.3
	France	35.1	2.0	4.7	41.8
	India	53.2	61.4	7.4	122.0
	Colombia	54.5	3.9	0.4	58.8
	Sweden	66.8	2.2	10.2	79.3
	Spain	54.0	26.8	13.4	94.3
	Poland	49.6	0.7	13.8	64.1
	Netherlands	100.6	3.8	13.7	118.1
	Italy	51.1	8.5	13.8	73.5
	*Mean*	**60.9**	**10.0**	**12.1**	**83.0**
Biodiesel	USA	83.78	0.07	4.38	88.23
	Brazil	104.60	0.05	0.73	105.38
	France	67.58	1.52	9.02	78.13
	Germany	71.22	0.31	10.51	82.04
	Italy	77.38	4.41	6.13	87.92
	China	65.35	0.62	5.75	71.72
	Thailand	79.33	0.00	6.31	85.65
	Spain	96.28	10.04	4.93	111.26
	Poland	68.50	0.24	0.71	69.45
	UK	97.32	0.17	5.85	103.34
	Argentina	102.12	0.27	0.55	102.94
	Sweden	67.94	1.73	9.28	78.95
	Austria	78.35	0.47	8.17	86.99
	Colombia	76.67	0.02	3.64	80.33
	Indonesia	101.88	0.01	7.12	109.01
	Turkey	85.68	0.26	7.03	92.97
	Belgium	75.63	1.33	5.98	82.94
	Portugal	93.65	4.63	6.46	104.74
	Netherlands	88.83	0.60	5.14	94.58
	Canada	96.15	0.74	7.23	104.13
	Peru	61.68	0.00	6.57	68.25
	Denmark	85.26	0.27	5.92	91.45
	Czech Republic	71.56	1.67	26.36	99.59
	Finland	83.77	0.90	7.58	92.25
	Romania	132.59	0.20	5.02	137.82
	Greece	95.37	2.37	4.56	102.30
	Malaysia	93.69	0.00	4.09	97.78
	Slovakia	85.78	0.19	14.13	100.09
	India	98.27	0.49	5.71	104.48
	*Mean*	**85.7**	**1.2**	**6.7**	**93.6**
