# Supplementary material for: The water-land-food nexus of first-generation biofuels
Source: Sci Rep. 2016 Mar 3;6:22521. doi: 10.1038/srep22521 (PMC4776133; doi:10.1038/srep22521)
Supplement: Supplementary Information [file srep22521-s1.pdf]

The water-land-food nexus of first generation biofuels

Maria Cristina Rulli[1], Davide Bellomi[1], Andrea Cazzoli[1], Giulia De Carolis[1] and Paolo D'Odorico[2,3,*]

[1]Department of Civil and Environmental Engineering, Politecnico di Milano, Piazza Leonardo da Vinci 32, I-20133 Milan, Italy

[2] Department of Environmental Sciences, University of Virginia, 291 McCormick Rd., Charlottesville, VA 22903, USA

[3] National Socio-Environmental Synthesis Center, University of Maryland, Annapolis, MD 21401

* To whom correspondence should be addressed: paolo@virginia.edu

Supporting information

Additional Methods

Our estimates of biofuel production, consumption, and trade are based on data reported in the FAO Food Balance Sheets[1] for alcohol and vegetable oils for "other uses" (i.e., non-alimentary uses, mainly biofuel production[2,1]) as well as on a number of sources listed below (Table S1). This approach differs from the method used by[3], whose country-specific estimates of biofuel usage are based on data of gasoline and diesel consumption and on the expected percentage of biofuel blending.

Bioethanol is produced through the alcoholic fermentation of barley, maize, wheat, rye, sugar cane, sugar beet, and sweet sorghum (Fig. 1). The alcohol consumption data, reported as "other uses" in the Food Balance Sheets[1], include also ethanol use in green chemistry, pharmacological and cosmetic industry, and other uses related neither to food, nor bioenergy. Therefore the total amount of ethanol used for biofuel in each country was determined using other sources (Table S1) reporting country-specific total fuel bioethanol production and consumption (i.e. Eurostat database[4], US Energy International Administration[5], USDA-Foreign agricultural service-Global Agricultural Information Network,[6] Epure[7] , UK Department for Transport[8], French Environment and Energy Management Agency. (ADEME)[9], Swedish Energy Agency[10] , Italian Ministry of

Economic Development[11], Agência Nacional do Petróleo, Gás Natural e Biocombustíveis – ANP[12]).

The Food Balance Sheets[1] report for each country the relative contribution of bioethanol crops to alcohol and crop production for "other uses (i.e., not related to food), including both crops and alcohol available through domestic production and trade. Because the sources listed in Table S1 report the total bioethanol production without usually specifying the base crops (which are needed to calculate the water and land footprints of bioethanol), the relative contribution of bioethanol crops to bioethanol production is determined by partitioning the total values proportionally to the amounts of bioethanol crops available for other uses in each country, based on data from the Food Balance Sheets[1]. For some countries (Brazil, Canada, China, India, Colombia, Rep. Czech) we were able to refine this analysis using data on biofuel feedstock type and origin (USDA-GAIN[13,14,15,16,17,18])

Likewise, for the oils used in the biodiesel industry (rape-mustard seed oil, palm oil, and soybean oil) the Food Balance Sheets[1] report country-specific consumption data for "other uses". The vegetable oils for "other uses" are utilized not only for biofuels but also for the production of soap, cosmetics, and other non-food commodities[19]. To overcome this issue, we used the amount of vegetable oil for biodiesel, using data available in other sources reported in Table S1 (i.e. Eurostat database[4], US Energy International Administration[5], USDA-Foreign agricultural service-Global Agricultural Information Network[6], UK Department for Transport[8], French Environment and Energy Management Agency. (ADEME)[9], Swedish Energy Agency[10] , Italian Ministry of Economic Development[11], Agência Nacional do Petróleo, Gás Natural e Biocombustíveis – ANP[12]). The contribution of biodiesel crops to the total biodiesel amounts from these sources where determined proportionally to the values of biodiesel crops and vegetable oil available for other uses in each country, based on the Food Balance Sheets[1]. For some countries (i.e. Brazil, Colombia, Rep. Czech, Argentina, Italy) we could carry out a detailed analysis, based on data sets (USDA-GAIN[13,16,18,20, 21]) reporting the amount, type and origin of feedstock.

The European Union, the major biodiesel market[22], uses both locally produced rape and mustard seed oils, and imported palm and soybean oils [23, 24, 25]. In Asia, the biodiesel

industry relies mostly on palm oil from Malaysia and Indonesia, but it is overall not well developed [26, 27]. African countries contribute to the biodiesel industry as producers of oil crop feedstock but not as consumers of biodiesel[28]. South American countries produce biodiesel made primarily from soybean oil, of which Argentina and Brazil are the two main producers[25, 29,]. [30] To determine the impact of bioethanol and biodiesel consumption on food security, we also evaluate the water and land footprints of biofuel energy - defined as the amount of water and land required to produce one unit of biofuel energy, respectively. To do so, we use the biofuel production and water/land footprint estimates calculated as described below along with values of biofuel energy produced per mass of biofuel crop from Ref. 12. We also calculate the equivalent food crop calories that are "lost" to produce one unit of biofuel energy and determine the number of people that could be fed with all the crops and vegetable oils used to produce biofuels.

The water consumption of biofuels is calculated using water footprint values determined for each crop and oil by[31]. These values are country- (and crop-) specific, and include three components: green water (i.e., amount of rainwater used by the crop), blue water (amount of irrigation water transpired by the crop), and grey water (amount of water required to dilute nitrogen fertilizers and bring their concentrations within acceptable standards). We estimate the country-specific water footprints of bioethanol and biodiesel for the major (i.e., Top 90%) consumers by taking into account their imports. To this end, we investigate their trade relations and determine the geographical origin of water consumed for biofuels. This analysis is complicated by the fact that the FAO trade database [1] often reports as exporting countries major trade hubs (e.g., the Netherlands, Belgium, The United Arab Emirates, Singapore) that did not actually produce that commodity but re-exported it. We identify those instances by noting that a country cannot export more than what it produces. Thus, we use export and production data to distinguish exporters (i.e., countries producing the commodities they export) from re-exporters (i.e., countries importing the commodities they export), depending on whether their export is smaller or greater than a certain fraction of their domestic production, here taken equal to 33%. Our estimates of the water footprint of biofuels are overall insensitive to changes in this parameter within the 25%-50% range.

To evaluate the land footprint, we determine the land area required to produce one unit of biofuel energy using country-specific values of agricultural yields for each biofuel crop, based on data available for 2009-2013[1]. We account for crop and vegetable oil trade considering country-specific yields weighted by trade following the procedure described below for the water footprint.

We use import data from the FAOSTAT-Food Balance Sheets[1] and trade data from FAOSTAT-Trade[1] to determine the countries of origin. Because of some inconsistencies between these two datasets about the total import, we use total import values as reported in the FAO-Food Balance Sheets, and partition them among the exporting countries with the same proportions as in the FAO-Trade data[1]. We then assign local water footprint (WF) values to locally produced biofuel crops, while for imported crops we use the water footprint of the exporting country. In the case of re-exported crops we use the global average water footprint for the traded crops or oils. Notice that, unlike previous studies[3] here we investigate the water footprint of the aggregate of all the biofuel crops used for bioethanol or biodiesel production in each country, rather than the water footprint of a specific biofuel crop. This allows us to determine the overall appropriation of natural resources by biofuels.

To calculate the number of people who could be fed by biofuel crops, we use food calorie data for each edible crop or oil[32], and diets specific to both the producing and consuming countries (Table S2), based on the Food Balance Sheets[1]. We also refer to the case of a reference “balanced” diet (2700 kcal/day per capita, 80% vegetal and 20% animal)[33]. This analysis is more straightforward for bioethanol (the majority of biofuels produced worldwide) than for biodiesel because the starchy crops used for bioethanol production are staple aliments in human diets, while vegetable oils constitute a smaller fraction of the diet. Nevertheless, calories are here used as a metric to evaluate the impact of biofuels on food security. Thus, we determine the number of people who could be fed by biofuel crops based only on their caloric content and the per capita calories demand, without accounting for the viability of different dietary scenarios based on their macronutrient composition (e.g., protein vs carbohydrate content). Moreover, in this analysis we do not account of the byproducts of biofuel crops.

We next calculate the number of people that could have their food and energy demand met by the global cropland area, A. In this analysis we used per capita energy consumption (transport only), E, equal to 0.0143TJ/cap/yr and 0.0267TJ/cap/yr for the World and the E.U., respectively, which correspond to the 2,320,062 ktoe and 320,305 ktoe reported for the world and the E.U., in 2009, respectively[34]. We consider the average daily diet, D (expressed in amount of daily food calorie intake) for the World and the E.U., based on data from the Food Balance Sheets[1]. D includes both vegetable calories (Veg) and animal calories (An), D=Veg+An. We convert D into equivalent animal calories (D_e) calculated as D_e=Veg + c_{va} × An, with c_{va} being the vegetable feed calories-to-animal calories conversion factor[35]. Based on data reported in the Food Balance Sheets, we find that c_{va} =2.24 for the whole World and c_{va} =2.38 for the E.U. Thus we have that D_e=3,442 kcal/cap/d (World) and D_e=4,617 kcal/cap/d (E.U.). Notice that these values of c_{va} are lower than those reported by Pimentel[36] because they are based only on feed data reported in the Food Balance Sheets[1], i.e., considering only the grain fed share of animal food.

If we consider only temporary and permanent cropland and exclude fibers, we find that A=1.23x10^9 ha (based on FAO data[1]). If A is partly used for food (A_f) and partly for bioenergy (A_e) production (i.e., A=A_f+A_e), we can calculate the maximum size of the global population this land could support with different degrees of reliance on bioenergy. Globally, the average productivity of cropland is C_f=7.37 x 10^6 kcal/ha/y (based on Food balance sheets, including all crops used for food and feed and excluding crops for "other uses" 2009); we consider the current diet, D (in kcal/cap/d) as given by the Food balance sheet and convert it into equivalent vegetal calories (D_e) by multiplying the fraction of D contributed by animal food products by the vegetable calories required to produce those products using conversion factors determined from the Food Balance Sheets[37,35]. As noted, D_e is 4617 kcal/cap/d for the E.U. and 3442 kcal/cap/d for the World. The population P that can be fed by A_f is P=A_f C_f /(D_e x 365). Likewise, if we refer to the average per capita energy consumption for transport only, E, (E=0.0267 TJ/cap/yr in the E.U., and E=0.0146 TJ/cap/yr on average for the World [31]) and consider a degree of reliance on biofuels, b (fraction of fuel contributed by biofuels), using the average energy yields per unit area for bioethanol, C_e=0.1 TJ/ha (Table 3), we can relate A_e to P as P=A_e C_e/(b E). Thus we have that A= A_f+A_e = P[Dx365/C_f+bE/C_e]. Solving for P, we obtain

an expression for P as a function of the degree of the land footprints of food and biofuels and of the degree of the societal reliance on bioenergy.

REFERENCES

[1]Food and Agriculture Organization of the United Nations. *FAOSTAT Database*, available: http://faostat3.fao.org/faostat-gateway/go/to/download/FB/*/E [Accessed 22 Jan 2014]. (2009)

[2] Myers, R.L.& Myers, R. L.. *The 100 most important chemical compounds: a reference guide.* Westport, Conn.: Greenwood Press. p. 122. ISBN 0-313-33758-6 (2007).

[3] Gerbens-Leenes, P.W. & Lienden, A.R., et al., , Biofuel scenarios in a water perspective: the global blue and green water footprint of road transport in 2030. *Global Environmental Change* 22 (3), 764–775 (2010).

[4] Eurostat database (http://ec.europa.eu/eurostat/en/data/database), accessed on July 10th, 2015.

[5] US Energy International Administration (EIA) database (http://www.eia.gov/), accessed on July 15th, 2015.

[6] USDA-Forein agricultural service-Global Agricultural Information Network (http://gain.fas.usda.gov/Pages/Default.aspx), accessed on July 15th, 2015.

[7] EPURE statistics http://www.epure.org/resources/statistics

[8] UK department for Transport, RTFO 2012–2014 year four report. London, UK: Department for Transport.

[9] French Environment and Energy Management Agency (ADEME), statistics on biofuel.

[10] Swedish Energy Agency, Sustainable biofuel and bioliquids 2013, file:///Users/Cristina/Downloads/Sustainable%20biofuels%20and%20bioliquids%202013.PDF

[11] Italian Ministry of Economic Development, statistics on biofuel, http://www.sviluppoeconomico.gov.it/index.php/it/

[12] Agência Nacional do Petróleo, Gás Natural e Biocombustíveis – ANP, Statistic on biofuel, http://www.anp.gov.br/

[13] Biofuels annual-Brazil, USDA Foreign agricultural service GAIN Report Number BR14004.

[14] Biofuels annual-China USDA Foreign agricultural service GAIN Report Number CH14038.

[15] Biofuels annual-India, USDA Foreign agricultural service GAIN Report Number IN5079.

[16] Biofuels annual-Colombia, USDA Foreign agricultural service GAIN Report Number IN5079.

[17]Biofuels annual-Canada 2014, USDA Foreign agricultural service GAIN Report Number CA14109

[18] Biofuels annual-Czech Republic 2015, USDA Foreign agricultural service GAIN Report Number EZ1509

[19] The Global Land Project (GLP), *Contemporary land-use transition: the global oil palm expansion*, GPL Report no. 4 (2012).

[20] Biofuels annual-Argentina 2013, USDA Foreign agricultural service GAIN.

[21] Biofuels annual Overview-Italy 2015, Foreign agricultural service GAIN Report Number IT1526

[22] FAO-OECD, *Food and Agriculture Organization/Organization for Economic Co-operation and Development: Price Volatility in Food and Agricultural Markets: Policy Responses*. Food and Agriculture Organization of the United Nations, Rome, Italy (2011).

[23] The Global Land Project (GLP), Contemporary land-use transition: the global oil palm expansion, GPL Report no. 4 (2012).

[24] Gui, M. M., Lee, K. T. & Bhatia, S. Feasibility of edible vs. non-edible oil vs waste oil as biodiesel feedstock. *Energy* 33:1646-53 (2008).

[25] International Council on Clean Transportation, *Vegetable oils market and biofuel mandate* (2013)

[26] China Integrated Energy Inc., *FORM 10-K*, available: http://www.getfilings.com/sec-filings/100331/China-Integrated-Energy-Inc_10-K/ [Accessed 24 Feb 2014]. (2010).

[27] Zhou A, & Thomson E., The development of biofuels in Asia. *Appl. Energy* ;86:11–20 (2009).

[28] Amigun, B., Sigamoney, R. &von Blottnitz, H., Commercialization of biofuel industry in Africa: a review. *Renewable and Sustainable Energy Reviews* 12, 690–711 (2008).

[29] Janssen, R., Rutz, D. D. (2011), Sustainability of biofuels in Latin America: Risks and opportunities. Energy Policy 2011;39:5717–25.

[30] FAO-OECD, *OECD-FAO Agricultural Outlook 2013-2022*, OECD publishing (2013).

[31] Mekonnen, M.M. & Hoekstra, A.Y. *National water footprint accounts: the green, blue and grey water footprint of production and consumption*, Value of Water Research Report Series No. 50, UNESCO-IHE, Delft, the Netherlands (2011).

[32] D'Odorico P., Carr J.A., Laio F., Vandoni S. & Ridolfi L., Feeding humanity through the food and water trade networks. *Earth's Future,* 2, doi:10.1002/2014EF000250 (2014).

[33] Porkka, M., Kummu M., Siebert S. & Varis O., From food insufficiency towards trade dependency: A historical analysis of global food availability, *PLoS One*, *8*(12), e82714, doi:10.1371/journal.pone.0082714 (2013).

[34] International Energy Agency, IEA database, [available at http://www.iea.org/statistics/statisticssearch/].

[35] Davis K.F., D'Odorico P., Rulli M.C., Moderating diets to feed the future, *Earth's Future*, 2, 10, 559–565 (2014).

[36] Pimentel, D. & Pimentel M.H., *Food, Energy, and Society,* 3rd ed., CRC Press, Boca Raton, Fl (2008).

[37] Rulli, M. C. & D'Odorico P. (2014), Food appropriation through large scale land acquisitions. *Environ. Res. Lett.*, **9**, 064030, doi:10.1088/1748-9326/9/6/064030.

Table S1 Data sources uses for total bioethanol and biodiesel production and consumption in each country in 2013.

	Country	Sources
Bioethanol	USA	2
	Brazil	2,3,9,13
	Canada	2,3,15
	China	2,3,10
	Germany	1,4,14
	UK	1,5,4
	France	1,6,4
	India	2,3,11
	Colombia	2,3,12
	Sweden	1,7,4
	Spain	1,4
	Poland	1,4
	Netherland	1,4
	Italy	1,3,8
Biodiesel	USA	2
	Brazil	2,3,9,13
	France	1,6,14
	Germany	1,3,14
	Italy	1,3,8,14,19
	China	2,3,10
	Thailand	2,3,18
	Spain	1,3,14
	Poland	1,3,14
	UK	1,5,14
	Argentina	2,3,16
	Sweden	1,7,14
	Austria	1,3,14

	**Colombia**	**2,3,12**
	**Indonesia**	**2,3**
	**Turkey**	**1,3**
	**Belgium**	**1,3,14**
	**Portugal**	**1,3,14**
	**Netherlands**	**1,3,14**
	**Canada**	**2,3,15**
	**Peru**	**2,3**
	**Denmark**	**1,3,14**
	**Czech Rep.**	**1,3,17**
	**Finland**	**1,3,14**
	**Romania**	**1,3,14**
	**Greece**	**1,3,14**
	**Malaysia**	**2**
	**Slovakia**	**1,3,14**
	**India**	**2,3,11**

[1] Eurostat database (http://ec.europa.eu/eurostat/en/data/database), accessed on July 10th, 2015.
[2] US Energy International Administration (EIA) database (http://www.eia.gov/), accessed on July 15th, 2015.
[3] USDA-Forein agricultural service-Global Agricultural Information Network (http://gain.fas.usda.gov/Pages/Default.aspx), accessed on July 15th, 2015.
[4] EPURE statistics http://www.epure.org/resources/statistics
[5] UK department for Transport, RTFO 2012–2014 year four report. London, UK: Department for Transport.
[6] French Environment and Energy Management Agency (ADEME), statistics on biofuel.
[7] Swedish Energy Agency, Sustainable biofuel and bioliquids 2013, file:///Users/Cristina/Downloads/Sustainable%20biofuels%20and%20bioliquids%202013.PDF
[8] Italian Ministry of Economic Development, statistics on biofuel, http://www.sviluppoeconomico.gov.it/index.php/it/
[9] Biofuels annual-Brazil 2014, USDA Foreign agricultural service GAIN Report Number BR14004.
[10] Biofuels annual-China 2015 USDA Foreign agricultural service GAIN Report Number CH14038.
[11] Biofuels annual-India 2015, USDA Foreign agricultural service GAIN Report Number IN5079.
[12] Biofuels annual-Colombia 2015, USDA Foreign agricultural service GAIN Report Number IN5079.
[13] Agência Nacional do Petróleo, Gás Natural e Biocombustíveis – ANP, Statistic on biofuel, http://www.anp.gov.br/
[14] Biofuels annual-UE28 2015, USDA Foreign agricultural service GAIN Report Number NL5028
[15] Biofuels annual-Canada 2014, USDA Foreign agricultural service GAIN Report Number CA14109
[16] Biofuels annual-Argentina 2013, USDA Foreign agricultural service GAIN.
[17] Biofuels annual-Czech Republic 2015, USDA Foreign agricultural service GAIN Report Number EZ1509
[18] Biofuels annual-Thailand 2015, USDA Foreign agricultural service GAIN Report Number TH5085
[19] Biofuels annual Overview-Italy 2015, Foreign agricultural service GAIN Report Number IT1526

Table S2. Country specific Diets

	kcal cap^{-1}day^{-1}	kcal$_{veg}$ cap^{-1}day^{-1}	kcal$_{an}$ cap^{-1}day^{-1}
Austria	3800	2663	1137
Belgium	3721	2595	1126
Brazil	3173	2436	737
Canada	3399	2506	893
China	3036	2342	694
Colombia	2717	2181	536
Czech Republic	3305	2427	878
Denmark	3378	2085	1293
France	3531	2348	1183
Germany	3549	2468	1081
India	2321	2113	208
Italy	3627	2694	933
Japan	2723	2158	565
Mexico	3146	2495	651
Netherlands	3261	2194	1067
Poland	3392	2473	919
Portugal	3617	2563	1054
Romania	3487	2568	919
Russian Federation	3172	2395	777
Spain	3239	2413	826
Sweden	3125	2055	1070
Thailand	2862	2547	315
United Kingdom	3432	2425	1007
United States of America	3688	2675	1013

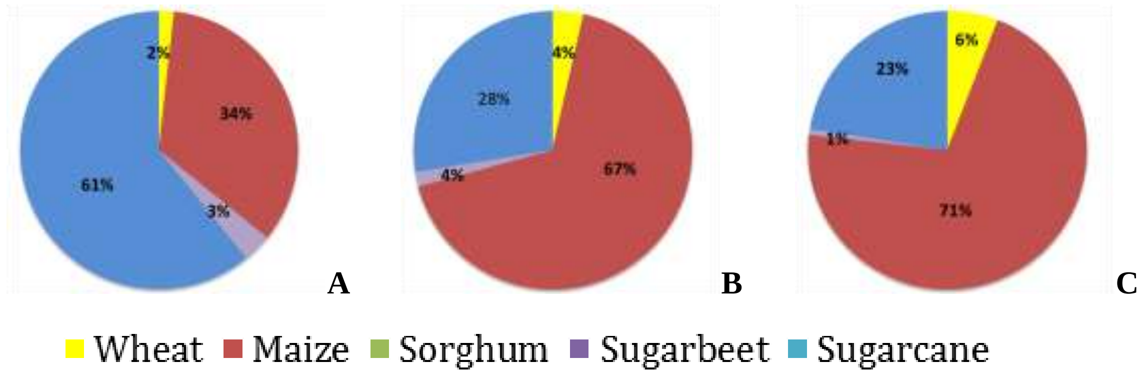

Figure S1. A) Proportion of crop mass contributing to the global bioethanol production; B) proportion of bioethanol produced using different crop types; C) Water consumed in bioethanol production.

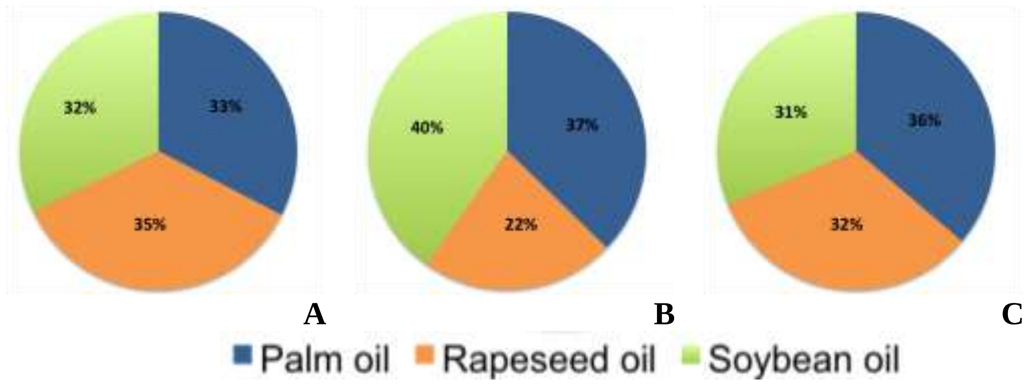

Figure S2. Relative importance of the consumption of biodiesel (A), crop biomass (B) and water (C) for the main crops used for biodiesel production.

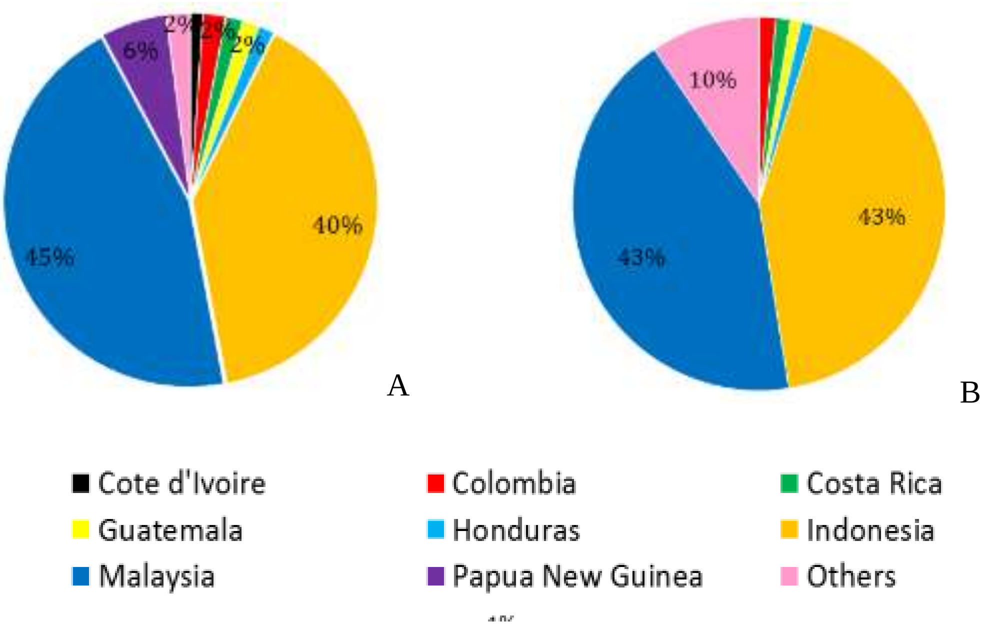

Figure S3. Palm oil imports for biodiesel production by OECD+EU27 countries (A) and the associated virtual water imports (B).
